# High burden of malaria and anemia among tribal pregnant women in a chronic conflict corridor in India

**DOI:** 10.1186/s13031-017-0113-1

**Published:** 2017-06-20

**Authors:** Gustavo Corrêa, Mrinalini Das, Rama Kovelamudi, Nagendra Jaladi, Charlotte Pignon, Kalyan Vysyaraju, Usha Yedla, Vijya Laxmi, Pavani Vemula, Vijaya Gowthami, Hemant Sharma, Daniel Remartinez, Stobdan Kalon, Kirrily de Polnay, Martin De Smet, Petros Isaakidis

**Affiliations:** 1 Médecins sans Frontières, Bhadrachalam, Khammam District, Telangana, 507111 India; 2 Government Hospital, Bhadrachalam, Khammam District, Telangana, 507111 India; 3 Médecins Sans Frontières, Brussels, 46, Rue de l’Arbre Bénit, 1050 Brussels, Belgium; 4 Operational Research Unit, Médecins Sans Frontières, Luxembourg, 68, rue de Gasperich, L-1617 Luxembourg, Belgium

**Keywords:** Malaria, Anemia, Antenatal care, Rapid diagnostic test

## Abstract

**Background:**

With more than 200 million cases a year, malaria is an important global health concern, especially among pregnant women. The forested tribal areas of Andhra Pradesh, Telangana and Chhattisgarh in India are affected by malaria and by an on-going chronic conflict which seriously limits access to health care. The burden of malaria and anemia among pregnant women in these areas is unknown; moreover there are no specific recommendations for pregnant women in the Indian national malaria policy. The aim of this study is to measure the burden of malaria and anemia among pregnant women presenting in mobile clinics for antenatal care in a conflict-affected corridor in India.

**Methods:**

This is a descriptive study of routine programme data of women presenting at first visit for antenatal care in Médecins sans Frontières mobile clinics during 1 year (2015). Burden of malaria and anemia were estimated using rapid diagnostic tests (SD BIOLINE® and HemoCue® respectively).

**Results:**

Among 575 pregnant women (median age: 26 years, interquartile range: 25-30) 29% and 22% were in their first and second pregnancies respectively. Mid-Upper Arm Circumference (MUAC) was below 230 mm in 74% of them. The prevalence of anemia was 92.4% (95% Confidence Intervals (CI): 89.9–94.3), while severe anemia was identified in 6.9% of the patients. The prevalence of malaria was 29.3% (95%CI: 25.7–33.2) with 64% caused by isolated *P. falciparum*, 35% by either *P. falciparum* or mixed malaria and 1% by either *P. vivax*, or *P.malariae* or *P. ovale*. Malaria test was positive in 20.8% of asymptomatic cases. Malaria was associated with severe anemia (prevalence ratio: 2.56, 95%CI: 1.40–4.66, *p* < 0.01).

**Conclusions:**

Systematic screening for malaria and anemia should be integrated into maternal and child health services for conflict affected populations in highly endemic tribal areas. Interventions should include the use of rapid diagnostic test for all pregnant women at every visit, regardless of symptoms. Further studies should evaluate the impact of this intervention alone or in combination with intermittent malaria preventive treatment.

## Background

Globally, more than 200 million cases of malaria occur every year with an estimated half a million deaths. The South-East Asia region contributes the second largest number of cases in the world and India alone is estimated to have between 10 and 26 million cases. One fifth of India’s population lives in high transmission areas (more than 1 case per 1000 population) and *Plasmodium falciparum* is estimated to be responsible for 66% of cases in the country [1].

Malaria is an important threat to pregnant women, with increased risk for both the mother and the newborn, especially in the first and second pregnancies and when caused by *P. falciparum* [2]. The risks involved are fetal death, prematurity, low birth weight, and severe maternal anemia among others. International guidelines recommend prevention and treatment for malaria and anemia for pregnant women in endemic areas [3, 4], but, it has not been included in the recently published Indian national policy for malaria elimination [5].

The forested areas of Chhattisgarh, Andhra Pradesh and Telangana are highly endemic for malaria (by both *P. vivax* and *P. falciparum*), but the prevalence of malaria in pregnant women in this area is not yet known. Different studies show very different figures ranging from 1.3% [6] to 20.6% [7], depending on the design and settings. However, according to our knowledge, no study has yet been performed in the tribal areas to estimate the prevalence of malaria among pregnant women regardless of symptoms.

An on-going chronic conflict between Maoist groups, known as Naxalites, and government forces has caused significant displacement of the tribal populations and has limited their access to even the most basic health services [8]. From 2005 to June 2016, more than 7000 people have been killed, with more than one third of these casualties in the Chhattisgarh state [9]. Since 2006, Médecins sans Frontières (MSF) is present in the region providing primary health care as well as antenatal care (ANC) close to the communities through weekly mobile clinics (MC).

The aim of this study was to determine the burden of malaria and anemia among tribal pregnant women in this chronic conflict area in order to inform policy and practice that would contribute to serve this population more effectively. The primary objective was to determine the prevalence of malaria among women presenting for ANC care at the MSF MC. Secondary objectives were 1) to describe clinical and demographic characteristics of pregnant women and their association with malaria infection; 2) to determine the proportion of *P. falciparum* infections among pregnant malaria patients in the area; 3) to determine the prevalence of anemia in pregnant women and to describe associations between malaria infection and anemia.

## Methods

### Study design and study population

This was a descriptive study of routinely collected data. All women presenting at first visit for ANC care in any of our MC in forested areas of Chhattisgarh, Andhra Pradesh and Telangana during 1 year (from January to December 2015) were included in the study. Women presenting for ANC care in this area were predominantly from the Koya or the Gotikoya ethnic groups.

### MSF programme description

The MSF intervention in the area includes weekly MC in conflict affected areas, as close to the communities as possible. The programme performs 12 different MC per week, returning to the same areas every week. The MC are usually located in an open area in the proximity of identified tribal settings. The locations of the MC are decided based on the identification of poor health access locations and after a careful security assessment. The MC provides general practitioners consultations, ANC and post-natal care consultations, health promotion, laboratory tests, and drug dispensation. All services are free of charge. In some locations an outreach team is able to extend treatment to the community level through the use of community health workers. Diagnosis and treatment for malaria in pregnancy are part of the intervention. Rapid Diagnostic Tests (RDT) for malaria and anemia are performed in every ANC visit and Quinine is the treatment provided for malaria if the diagnosis is made in the first trimester of pregnancy or Artemether and Lumefantrine (AL) if the diagnosis is made in the second or third trimesters. Long lasting insecticidal nets (LLIN) are distributed for all pregnant women, but no intermittent preventive treatment (IPT) has been provided.

### Diagnostic methods and operational definitions

For estimating the prevalence of malaria among women presenting to ANC for the first time, the proportion of positive cases measured by a RDT - SD BIOLINE® 05FK60 - at the first visit was considered. This RDT diagnoses the presence of two antigens for malaria: HRP-II present exclusively in *P. falciparum* infected patients and pan pLDH present in all forms of malaria (*P. falciparum*, *P. vivax*, *P. ovale*, *P.malariae*). Thus, if only HRP-II is positive, a diagnosis of isolated *P. falciparum* is made; if only pan pLDH is positive, a diagnosis of either *P. vivax* or *P. ovale* or *P.malariae* is made; and if both HRP-II and pan pLDH are positive a diagnosis of *P.falciparum* is made, with the possible diagnosis of mixed malaria. The co-infection of *P.falciparum* with one or more other forms of malaria (*P. vivax* or *P. ovale* or *P.malariae*) was used as a definition of mixed malaria. While the malaria treatment of pregnant women in the area did not change based on the different parasites diagnosed, the prevalence analysis of each antigen was presented by its epidemiological interest and relevance as a predictor of poor maternal and birth outcome.

For estimating the prevalence of anemia among women presenting for ANC for the first time, measurements of hemoglobin based on another RDT - HemoCue® Hb 301 - were used, at the first visit. The HemoCue is a very accurate diagnostic method when compared to a reference venous blood hematology analyser (Paired *t*-test *p* < 0.0001) [10]. The levels of hemoglobin used for defining anemia were the ones recommended by the World Health Organization (WHO) for pregnant women [11]. Thus, anemia cases were categorized as mild (10.0–10.9 mg/dl), moderate (7.0–9.9 mg/dl) and severe (<7.0 mg/dl). The prevalence of HIV was also estimated using an RDT - First Response® - measuring the presence of HIV 1 and 2. For HIV results, tests performed at any ANC visit were considered.

Mid-Upper Arm Circumference (MUAC) in mm was used to assess the nutritional status of pregnant women. Low MUAC levels (below 230 mm) is a well known predictor of poor birth outcomes, including low birth weight, preterm labour and intra-uterine growth retardation as well as poor maternal outcomes, including anemia and postpartum endometritis-myometritis, and it can also be used for enrolment in nutritional programmes in humanitarian contexts [12, 13]. The axillary temperature over 38 ° C at presentation was considered for the symptom *measured fever*. For *history of fever*, any self reported history of fever or symptoms of fever like sweating, shivering, headache and body pain in the last 2 week was considered. If no symptom has been registered in the ANC card, the case was considered asymptomatic.

### Data collection and analysis

Data were extracted from the ANC cards used in the MC and single entered in an Excel 2010 (Microsoft®) database, using data validation schemes for quality assurance. The data were then migrated to SPSS 18.0 (IBM®) for analysis. The association between the outcome of interest and selected variables was performed using prevalence ratios and Pearson Chi-square tests for categorical data. For continuous data, t–test for independent samples was used for comparing means between two groups – as was the case with hemoglobin – and Pearson’s r –ranging between -1 and +1, where +1 is total positive linear correlation, 0 is no linear correlation, and −1 is total negative linear correlation – for the correlation between hemoglobin and MUAC. Confidence interval (CI) of proportions were estimated using mid-P exact test on OpenEpi.com [14].

## Results

In total, 575 pregnant women attended a first ANC consultation on 300 different MC performed in 13 different settings in 2015 and were included in the study. The median age was 26 years (inter quartile range: 25-30) and overall, 29% of them were in their first pregnancy, and 22% were in their second one. Consultations during the first trimester of pregnancy corresponded to about one quarter of them, and the majority of consultations were for symptomatic patients (54%). There were 887 live births identified from previous pregnancies of which 18.3% were reported dead in the first year of life. Regarding the nutritional status of women, among those with MUAC evaluation (560), most of them were below 230 mm (74%). Table 1 summarizes the study population characteristics.

**Table 1:** Characteristics of pregnant women from Chhattisgarh, Andhra Pradesh and Telangana attending first visit of ANC care in MSF Mobile Clinics in 2015

Description (*n* = 575)	Total	% or range
**Median age** (**range**)	**26**	(**15**–**48**)
Less than 20	20	3.5%
20–24	108	18.8%
25–29	275	47.9%
30–34	133	23.2%
35–39	33	5.7%
More than 39	5	0.9%
**Median previous pregnancies** (**range**)	**2**	(**1**–**11**)
**Median pregnancy month** (**range**)	**5**	(**1**–**9**)
First trimester	149	25.9%
Second trimester	216	37.6%
Third trimester	209	36.3%
**Median Weight** (**range**)	**42**	(**25**–**80**)
**Median MUAC** (**range**)	**220**	(**106**–**368**)
Less than 186 mm	11	2.0%
186–209	105	18.7%
210–230	300	53.6%
More than 230	144	25.7%
**Asymptomatic patients**	**263**	**45.7** %

Missing values were generally uncommon for age (0.2%), gestational age (0.2%), previous pregnancies (1.2%), hemoglobin results (2%), malaria results (2%) and MUAC results (2.6%). The only exam with a significant number of missing values was the HIV test (26.6%). The missing results were fairly distributed across all locations, except for hemoglobin, that was concentrated on three different MC, ranging from 4 to 16.6% of all patients attended on those locations. Only one patient was missing both hemoglobin and malaria results and only 3 patients were missing both MUAC and hemoglobin results.

The prevalence of malaria was 29.3% (95%CI:25.7–33.2), with 64% of them diagnosed by HRP-II only (isolated *P. falciparum*), 1% by pan pLDH only (either *P. vivax* or *P.malariae* or *P. ovale*), and 35% by both HRP-II and pLDH (either isolated *P. falciparum or mixed malaria*). *P.falciparum* was diagnosed in 99% of all malaria cases. Table 2 summarizes the main study outcomes.

**Table 2:** Malaria and anaemia in pregnant women from Chhattisgarh, Andhra Pradesh and Telangana attending first visit of ANC care in MSF Mobile Clinics in 2015

Exams performed (*n* = 575)	Total tested	Positive cases	%	CI (95%)
**Malaria positive**	**563**	**165**	**29.3**	(**25.7**–**33.2**)
HRP-II only (isolated *P.falciparum*)	563	106	18.8	(15.8–22.2)
pLDH only (either *P. vivax* or *P. ovale* or *P. malariae*)	563	1	0.2	(0.0–0.9)
Both (either isolated *P.falciparum* or mixed malaria)	563	58	10.3	(8.0–13.0)
**Anemia**	**563**	**520**	**92.4**	(**89.9**–**94.3**)
Severe (<7 mg/dl)	563	39	6.9	(5.0–9.2)
Moderate (7–9.9 mg/dl)	563	379	67.3	(63.4–71.1)
Mild (10–10.9 mg/dl)	563	102	18.1	(15.1–21.5)
**HIV**	**422**	**2**	**0.5**	(**0.1**–**1.6**)

**Fig. 1:**
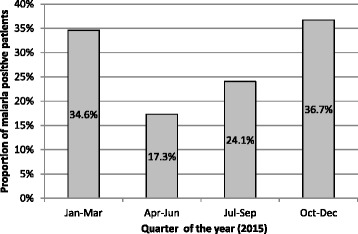
Prevalence of malaria in pregnant women from Chhattisgarh, Andhra Pradesh and Telangana attending first visit of ANC care in MSF Mobile Clinics in 2015

The prevalence of malaria varied according to the period of the year in which the pregnant women had their first visit (Fig. 1). Higher prevalence was observed from October to November (36.7%), and lower prevalence from April to June (17.3%).

Among the main symptoms reported by the patients, only fever (measured, history or both) was positively associated with malaria with statistical significance. The prevalence ratio of fever for malaria positive cases was 4.05 (CI95%: 3.3–5.0), 2.65 (CI95%: 1.9–3.7) for only HRP-II positive (isolated *P. falciparum*) and 9.02 (CI95%: 5.5–14.8) for both HRP-II and pLDH positive (either isolated *P. falciparum* or mixed malaria). The prevalence of malaria among asymptomatic cases was about half the prevalence of symptomatic cases, corresponding to about 20.8% in the first group and 36.6% in the second (Table 3).

**Table 3:** Association between malaria and clinical symptoms of pregnant women from Chhattisgarh, Andhra Pradesh and Telangana attending first visit of ANC care in MSF Mobile Clinics in 2015

Symptoms (*n* = 563)	Malaria Positive			Only HRP-II (isolated *P.falciparum*)			Both HRP-II and pLDH (either isolated *P.falciparum* or mixed malaria)		
	Prevalence ratio ^a^	CI (95%)	p (χ^2^) ^b^	Prevalence ratio ^a^	CI (95%)	p (χ^2^) ^b^	Prevalence ratio ^a^	CI (95%)	p (χ^2^) ^b^
1. Measured Fever	3.84	(3.13–4.79)	<0.01	2.45	(1.73–3.48)	<0.01	8.21	(5.17–13.02)	<0.01
2. History of fever	2.28	(1.62–3.21)	<0.01	2.28	(1.34–3.89)	0.01	2.32	(1.03–5.22)	0.05
3. Fever (history or measured)	4.05	(3.27–5.02)	<0.01	2.65	(1.90–3.69)	<0.01	9.02	(5.49–14.80)	<0.01
4. Weakness	0.86	(0.52–1.43)	0.55	0.90	(0.46–1.73)	0.74	0.81	(0.39–2.15)	0.67
5. Headache	1.33	(0.80–2.20)	0.29	1.24	(0.60–2.55)	0.57	1.53	(0.60–3.90)	0.38
6. Body pain	0.61	(0.42–0.87)	<0.01	0.61	(0.38–0.97)	0.03	0.62	(0.32–1.19)	0.14
7. Dizziness	0.42	(0.11–1.54)	0.13	0.66	(0.18–2.43)	0.51	N/A	N/A	0.17
8. Nausea or vomiting	0.69	(0.39–1.22)	0.18	0.53	(0.23–1.24)	0.12	1.01	(0.42–2.41)	0.98
9. Asymptomatic	0.57	(0.43–0.75)	<0.01	0.74	(0.52–1.05)	0.09	0.30	(0.16–0.56)	<0.01

^a^Unadjusted ratios; ^b^Pearson chi-square

The prevalence of anemia in the study population was 92.4% (95%CI: 89.9–94.3), 7.5% was severe, 72.9% moderate and 19.6% mild. The mean hemoglobin level was 9.0 mg/dl (standard deviation: 1.44). HIV prevalence was 0.5% (95%CI: 0.1–1.6), but HIV results were not available in more than a quarter of patients.

Malaria was associated with severe anemia with statistical significance (prevalence ratio: 2.56 CI95%: 1.4–4.7, *p* < 0.01), but not with anemia (prevalence ratio: 1.05 CI95%: 1.0–1.1, *p* = 0.08). Positive malaria cases had a mean hemoglobin level of 0.6 mg/dl (CI95%: 0.86–0.33, *p* < 0.01) lower than negative cases (Table 4) and a very poor correlation was seen between MUAC and hemoglobin levels (Pearson’s R: 0.06, *p* = 0.15).

**Table 4:** Association between malaria and anemia among pregnant women from Chhattisgarh, Andhra Pradesh and Telangana attending first visit of ANC care in MSF Mobile Clinics in 2015

	Anemia			Mild anemia			Moderate anemia			Severe anemia		
	Prevalence ratio^a^	CI (95%)	p (χ^2^) ^b^	Prevalence ratio^a^	CI (95%)	p (χ^2^) ^b^	Prevalence ratio^a^	CI (95%)	p (χ^2^) ^b^	Prevalence ratio^a^	CI (95%)	p (χ^2^) ^b^
**Malaria positive**	**1.05**	(**1.00**–**1.10**)	**0.08**	**0.64**	(**0.41**–**0.99**)	**0.04**	**1.06**	(**0.94**–**1.20**)	**0.34**	**2.56**	(**1.40**–**4.66**)	<**0.01**
HRP-II only^c^	1.04	(0.98–1.09)	0.26	0.81	(0.50–1.32)	0.39	1.00	(0.86–1.16)	0.98	2.15	(1.15–4.05)	0.02
Both HRP-II and pLDH^d^	1.05	(0.99–1.10)	0.25	0.46	(0.20–1.08)	0.06	1.13	(0.96–1.33)	0.19	1.94	(0.90–4.18)	0.09

Malaria and anemia (*n* = 552)

^a^Unadjusted ratios; ^b^Pearson chi-square; ^c^isolated P.falciparum; ^d^either isolated P.falciparum or mixed malaria

## Discussion

### Implications for policy and practice

This study confirms the high burden of malaria among tribal pregnant women in a chronic conflict corridor in India. It also shows that the burden of malaria among pregnant women varies according to seasonal patterns, being even more relevant during the high malaria season from October to March. In addition, it indicates that malaria infections among pregnant women in this area present a high risk, as the risk of malaria infection in pregnancy is especially high during first and second pregnancies [15]. More than 50% of pregnant women presenting for ANC care in this setting are in the first and second pregnancies.

Most malaria cases identified (99%) were caused by *P. falciparum* (isolated or mixed with other forms of malaria) which may add substantial risk to the patients. This finding is aligned with the results of other studies on malaria transmission dynamics made in this region in the past decades. Those studies show that in forested tribal areas in this region, malaria transmission seems to be perennial [16], and increasingly dominated by *P. falciparum* species [17–20], while *P. vivax* seems to be the predominant species in villages and urban settings [21].Moreover, the burden of malaria in India seems to be unequally concentrated in tribal areas, as a recent study performed with nationally representative data recently demonstrated [22]. This study demonstrates that the 8% Indian population living in districts with large tribal areas bear 46% of all malaria cases, 70% of all *P. falciparum* cases and 47% of all malaria deaths in the country. The fact that *P. falciparum* is the predominant species in tribal areas may add substantial risk to pregnant women. *P. vivax* is theoretically less dangerous for this group of patientsbecause it does not cytoadhere in the placenta, yet it may also cause low birth weight and maternal anemia [15]. The fact that the RDT used did not detect the pan pLDH antigen as often as the *P. falciparum* (HRP-II) one, does not necessarily means that *P*. vivax is not a common cause of malaria in the area but in this study, we could not isolate *P.vivax* from other forms of malaria.. In some areas of the Indian subcontinent, *P. vivax* malaria exhibit two seasonal peaks, the first of which is dominated by relapses (January to June) and the second one is shared with *P. falciparum* in periods of high transmission after the monsoon rain (July–Nov) [23]. The seasonal pattern of *P. vivax* and its consequences for pregnant women should be further evaluated in this area by future studies. The only symptom strongly associated with malaria and with statistical significance was fever (either history of fever, measured fever, or both). It could be then used in diagnostic algorithms for increasing the pre-test probability, as it is already recommended in the national guidelines [24]. Studies suggest that traditional healers may play an important role on the treatment of fever in tribal communities [25], and efforts to reduce malaria burden in tribal areas should consider partnering and promoting health education activities with traditional healers for the management of fever cases. There is also a high prevalence of malaria in asymptomatic pregnant women (20.8%), which is aligned with other studies performed in the area [26, 27]. It may possibily be attributed to the fact that malaria infections in healthy adults, including pregnant women, in moderate to high transmission areas rarely result in fever [15]. Therefore, elimination of malaria is highly unlikely if diagnostic strategies do not include asymptomatic patients, because they will remain a reservoir of parasites contributing to the spread of the disease from one malaria season to the next. This seems to represents an opportunity for using ANC services in tribal areas for targeted approaches, and intermittent screening and treatment (IST), as recommended by WHO [28], using RDT screening and artesimin-based combinations therapies during ANC care seems to a reasonable option. Despite of increased programmatic costs, it has been demonstrated that IST is a safe and effective intervention to pregnant women seeking malaria care in moderately high transmission areas [29].Nevertheless, the national guideline for diagnosis and treatment of malaria do not consider targeted approaches for screening of vulnerable groups such as pregnant women or tribal areas [24].

Another intervention opportunity is the use of IPT for pregnant women. The use of Sulfadoxine-Pyrimethamine (SP) as early as possible from the second trimester of pregnancy onwards with subsequent doses at least 1 month apart up to the time of delivery is recommended by WHO in areas of moderate to high malaria transmission in Africa, although the exact level of malaria transmission to indicate it is not yet clear [30]. IPT is not yet recommended outside Africa because there is insufficient evidence for general recommendations in other areas. Nevertheless, a recent meta-analysis clearly demonstrated the benefits of IPT in maternal and child health in malaria endemic areas, mainly in first and second pregnancies [2]. In addition, IPT remains an effective intervention in preventing the adverse consequences of malaria on maternal and fetal outcomes, even in locations where there is a high prevalence of SP resistance genetic mutations on *P. falciparum* parasites, leading to in vivo resistance [30], as it seems to be the case in India [31]. We suggest the use of IPT in highly endemic areas in India, specifically for isolated tribal communities where access to health care has been compromised by conflicts, on the provision that it is submitted to subsequent evaluation on maternal and infant health outcomes, like maternal anemia and low birth weight. Mathematical modelling could help to understand the malaria transmission threshold below which IPT is no longer cost-effective.

The nutritional level of pregnant women in the study area seems to be very poor. Low MUAC levels have been demonstrated to generate low birth weight and preterm labor, along with anemia and post-partum endometritis in a recent meta-analysis [13]. Levels of MUAC below 230 mm were consistently statistically significant for poor birth outcomes in previous studies [12], and most of our patients are included in this group. A poor nutritional status seems to pose an even higher risk to pregnant women with malaria and their newborns [32]. However, a previous study showed that nutritional supplements do not seem to impact the immunological response to malaria of undernourished pregnant women with less than 20 weeks of pregnancy [33]. Perhaps an earlier supplementation could be more effective and should be further investigated. The high level of under-nutrition could help to explain the high level of anemia in the area, but the poor correlation between MUAC and hemoglobin levels suggests that nutritional status is unlikely to play a major role in hemoglobin levels.

It was also not possible to establish a clear association between malaria and anemia, perhaps because the burden of anemia in the study area was very high (92.4%) and impossible to justify with only malaria active cases. Nevertheless, the association of malaria with mean hemoglobin level was small (0.6 mg/dl), but statistically significant. Malaria seems to be also associated with severe anemia with a prevalence ratio of 2.56. This finding is consistent with other studies which have already demonstrated the association of malaria with severe anemia in pregnancy [15, 18]. This suggests that malaria active cases may not be the cause for most anemia cases, but it seems to contribute to the severity of some of them. Malaria infection diagnosed by both HRP-II and pLDH were not significantly associated with severe anemia, but this unusual finding may be due to a reduced sample size of the subgroup analysis. Improving access to malaria prevention, care and treatment could potentially reduce the need of hospitalisations and improve maternal health; however other causes of anemia may be playing an important role in the morbidity of pregnant women. Different studies have suggested a high prevalence of hemoglobinopathies in central India, including sickle cell disease and thalassemia [34, 35]. Sickle cell anemia is especially high in patients from Chhattisgarh, with some studies suggesting a prevalence as high as 35% in some communities [36]. Parasitic infections [37], recurrent malaria and other chronic diseases [38] have been suggested as causes of anemia in other studies and should be further investigated.

The low prevalence of HIV infections demonstrates the low burden of AIDS in this area; however, the test was not performed in many of our patients. For few patients, the test was not performed because the team was unable to provide counselling and testing before the closing time of the clinic which had to be respected due to security constraints. Some of the patients opted out of the test while others preferred to perform the test in government settings. These alleged reasons for refusal demonstrate that there is HIV stigma in those communities, which should be addressed by health promotion interventions.

The results of this study also show that, close to the community level, malaria burden is much worse than previous facility-based studies could demonstrate. One study performed in governmental ANC clinics in two different districts of the state of Chhattisgarh involving rural and urban population showed a malaria prevalence of 1.3% [6]. Another study performed in the state of Jharkhand in different ANC clinics identified a malaria prevalence of 1.8% (53% *P. falciparum* and 37.2% *P. vivax* and 9.3% mixed infections), being four times higher in rural than urban areas [39]. Still in Jharkhand, a study performed in a semi-urban ANC and Delivery Unit clinic showed a malaria prevalence of 4.3% (87% caused by *P. vivax*). The majority of cases in this study (85%) were identified in asymptomatic patients. However, the authors stated that less than 60% of women in this state search the hospital for deliveries [27]. Another study suggested that, among tribal pregnant women in this area, the prevalence was 20.6%. However, this study had a targeted approach of testing only febrile patients with RDT [7]. In another study of tribal populations (not only pregnant women) in the district of Purulia in the state of West Bengal, among 1040 asymptomatic patients, 81 (8.4%) were positive for *P. falciparum* and only 4 cases were positive for *P. vivax* [26]. Our study shows a higher prevalence of malaria among pregnant women (29.3%) compared to all previous studies in this conflict area, perhaps because we offer easily accessible services, free of charge and close to the community level. Previous studies results could be influenced by a lack of access of disadvantaged population to the ANC clinics and efforts should be made to bring ANC services as close to the community level as possible.

### Integration with maternal and child health for malaria elimination

In recent years, India has made remarkable progress in the management of its malaria burden. Through its malaria control strategy, it has halved the annual number of cases from two million in 2000 to less than one million in 2013 and it is now committed to malaria elimination, endorsing the regional goal of an Asia-Pacific free of malaria by 2030, and joining the work of the Asia Pacific Leaders Malaria Alliance [40].

India’s programme uses a strategy based on use of RDT, artemisinin-based combination therapy, LLIN and indoor residual spraying. The strategy has been recently boosted by the use of community-based diagnostic testing and treatment performed by female volunteers or Accredited Social Health Activists, which receive performance-based incentives for conducting home visits, tracking fever cases and submitting blood slides to the community health centres, among other assigned tasks [40].

The recent history of successful disease control induced a new strategy shift and, aligned with international recommendations, India has issued its framework for malaria elimination until 2030 [5]. However, despite acknowledging the need of special interventions for high risk groups, including tribal populations, populations residing in conflict affected or hard-to-reach areas, nothing is mentioned about strategies for pregnant women or integration of malaria care in other programmes, such as maternal or child health programmes. This is not aligned with international recommendations, as WHO recommends malaria and maternal care integration in both the most recent malaria [41] and maternal care [42] policy papers. The high burden of malaria in pregnant women found in this study highlights the importance of the integration between malaria and maternal and child health programmes, if elimination of malaria is to be achieved until 2030.

### Limitations

This study has some limitations. According to some projections made in the project, it is estimated that around one quarter of the pregnant women in the area access the MC ANC services every year. Despite being placed close to the community level, and with some degree of community involvement, our study was still mainly facility-based, and most of the local population still only looks for MC services when they have health problems. This helps to explain the high level of symptomatic patients (54%) in our ANC service, and this could overestimate the prevalence of malaria and anemia. Nevertheless, the high level of malaria and anemia in asymptomatic patients reduces the impact of this limitation.In addition, the RDT used for malaria diagnosis (SD BIOLINE® 05FK60) has some limitations. Firstly, the sensitivity of the test may be reduced depending on some conditions. The overall sensitivity of the test is 99.7% for *P.falciparum* and 95.5% for non *P.falciparum* malaria according to its specifications. However, studies performed in programmatic conditions using a combination of microscopy and PCR as a gold standard showed that the sensitivity of the test may range from 83.3 to 100% [43–45]. One study in the Central African Republic showed a sensitivity of 69.6% for *P. falciparum* patients with a parasitemia below 100 parasites per micro litre of blood on microscopy [46]. In the case of this study region, where transmission seems to be moderate to high, and consequently parasitemia levels of patients may be generally low, there is a possibility that some cases have been missed. Considering its specificity, the test specification suggests a value of 99.5%.Nevertheless different studies on programmatic conditions shows that it may range from 86.8 to 98.9%[43–45], thus it is also likely that some false positives results also occurred. Secondly, as this RDT may take some time to become negative after the patient has been treated because of the persistence of HRP-II, in the case of a patient accessing recent treatment for malaria before knowing her pregnancy status, the test may also present a false positive result. However the low level of health access in the area suggests a very limited impact of this limitation. Thirdly, as the study has used RDT results based on common antigen for different *Plasmodium* species, it was impossible to isolate the burden of isolated *P. falciparum* infections from mixed malaria. This limitation has less importance in pregnant women, as the clinic protocol does not change depending on the type of malaria and primaquine cannot be offered to pregnant women. It was also impossible to separate the burden of *P. vivax* from *P.malariae* and *P. ovale*. Although most studies in the area demonstrate the presence of only P.falciparum and P.vivax; *P.ovale* was also recently isolated in the Chhattisgarh area and its burden is not yet known [47].

Finally, this was a retrospective study based on routinely collected data and it is possible that some clinical history data were missing from the ANC cards, which could compromise the analysis on asymptomatic cases. It is very hard to estimate the size of this effect, but the general low level of missing results of exams suggests that the data are, in general, of reasonable quality.

## Conclusions

This study has shown that the burden of malaria and anemia among tribal pregnant women in a chronic conflict setting in the states of Chhattisgarh, Andhra Pradesh and Telangana in India is very high. It also demonstrates that malaria is not associated with anemia, but it is with severe anemia. Most of the malaria infections in this area are caused by *P. falciparum*, either isolated or mixed with other forms of malaria and asymptomatic malaria cases are common. Malaria prevention and treatment for pregnant women in high endemic areas should be integrated in the national policy, especially for conflict affected areas with reduced access to health care. Interventions should include IST with systematic screening for all pregnant women with RDT in every visit regardless of symptoms..

Further studies should evaluate the impact of the suggested intervention alone, or in combination with IPT, the effect of the co-existence of malaria and poor nutritional status in pregnancy outcomes, and the other causes of anemia in this setting.

## Abbreviations

ANC: Antenatal Care
CI: Confidence Interval
HIV: Human Immunodeficiency VirusIPT - Intermittent Preventive Treatment
IST: Intermitent Screening and Treatment
MC: Mobile Clinic
MSF: Médecins sans Frontières
MUAC: Mid-Upper Arm Circumference
RDT: Rapid Diagnostic Test
SP: Sulfadoxine-Pyrimethamine
WHO: World Health Organization
